# Effect of Interleukin-7 on In Vitro Maturation of Porcine Cumulus-Oocyte Complexes and Subsequent Developmental Potential after Parthenogenetic Activation

**DOI:** 10.3390/ani11030741

**Published:** 2021-03-08

**Authors:** Dongjin Oh, Joohyeong Lee, Eunhye Kim, Seon-Ung Hwang, Junchul-David Yoon, Lian Cai, Mirae Kim, Gahye Kim, Hyerin Choi, Sang-Hwan Hyun

**Affiliations:** 1 Veterinary Medical Center and College of Veterinary Medicine, Laboratory of Veterinary Embryology and Biotechnology (VETEMBIO), Chungbuk National University, Cheongju 28644, Korea; rosecafes123@naver.com (D.O.); durubit@gmail.com (J.L.); iwsleh@nate.com (E.K.); ghkdsun@hanmail.net (S.-U.H.); jdyoon86@hanmail.net (J.-D.Y.); cailian005@nate.com (L.C.); kmr9309@naver.com (M.K.); tr_hyeip23@naver.com (G.K.); hyrin3642@naver.com (H.C.); 2 Institute of Stem Cell & Regenerative Medicine (ISCRM), Chungbuk National University, Cheongju 28644, Korea; 3 Graduate School of Veterinary Biosecurity and Protection, Chungbuk National University, Cheongju 28644, Korea

**Keywords:** in vitro maturation, porcine oocytes, developmental potential, interleukin-7, parthenogenetic activation

## Abstract

**Simple Summary:**

Oocyte-secreted factors play an essential role in oogenesis and fertility through bidirectional crosstalk between oocytes and somatic cells. Interleukin-7, known as an oocyte-secreted factor, has recently been shown to improve oocyte developmental competence through interaction with cumulus cells around the oocytes. This study aimed to investigate the effects of interleukin-7 on porcine cumulus-oocyte complexes during in vitro maturation. Our results showed that supplementation with interleukin-7 during in vitro maturation exerted beneficial effects on porcine oocyte meiotic maturation by upregulating antioxidant-related genes and enhanced the subsequent developmental potential of porcine embryos after parthenogenetic activation.

**Abstract:**

Interleukin-7 (IL-7) is a cytokine essential for cell development, proliferation and survival. However, its role in oocyte maturation is largely unknown. To investigate the effects of IL-7 on the in vitro maturation (IVM) of porcine oocytes, we analyzed nuclear maturation, intracellular glutathione (GSH) and reactive oxygen species (ROS) levels, and subsequent embryonic developmental competence after parthenogenetic activation (PA) under several concentrations of IL-7. After IVM, IL-7 treated groups showed significantly higher nuclear maturation and significantly decreased intracellular ROS levels compared with the control group. All IL-7 treatment groups exhibited significantly increased intracellular GSH levels compared with the control group. All oocytes matured with IL-7 treatment during IVM exhibited significantly higher cleavage and blastocyst formation rates after PA than the non-treatment group. Furthermore, significantly higher mRNA expression levels of developmental-related genes (*PCNA*, *Filia*, and *NPM2*) and antioxidant-related genes (*GSR* and *PRDX1*) were observed in the IL-7-supplemented oocytes than in the control group. IL-7-supplemented cumulus cells showed significantly higher mRNA expression of the anti-apoptotic gene *BCL2L1* and mitochondria-related genes (*TFAM* and *NOX4*), and lower transcript levels of the apoptosis related-gene, *Caspase3*, than the control group. Collectively, the present study suggests that IL-7 supplementation during porcine IVM improves oocyte maturation and the developmental potential of porcine embryos after PA.

## 1. Introduction

Porcine embryos derived from in vitro maturation (IVM) are useful for research regarding reproduction [1,2]. In particular, as pigs have an organ structure that is physiologically similar to that of humans, many studies use porcine embryos in the field of biomedical research [3,4,5]. To obtain consistent results in these fields, such as the production of transgenic disease models, the ability of the oocyte must be satisfied at an early stage of oogenesis [6,7]. The improvement in oocyte capacity is closely related to IVM. To improve the efficiency of porcine IVM, addition of various growth factors and other molecules to the IVM medium has been suggested [8,9,10]. Although these efforts have led to significant advances in the porcine IVM system, the in vitro matured oocytes exhibit lower developmental competence compared with matured oocytes in vivo, as the in vitro environment is not as optimum as in folliculogenesis [11].

Environmental factors around the oocyte play an important role in the developmental competence of the oocyte. For example, during folliculogenesis, bidirectional somatic cell–oocyte signaling is essential to simultaneously alter follicle development with oocyte maturation [12]. In particular, oocyte-secreted factors (OSFs), which are soluble growth factors secreted from the oocyte, play a critical role in oogenesis and fertility by regulating the functions of granulosa cells and cumulus cells (CCs) [13]. Among the OSFs, growth differentiation factor 9 (GDF9) and bone morphogenetic protein 15 (BMP15) are known to be vital for the initiation of primordial follicles and follicular development in many species [14]. In mice, deletion of *GDF9* and *BMP15* is detrimental to ovarian follicular development and fertility [15,16]. In addition, GDF9 and BMP15 co-treatment during IVM increases the embryonic development of porcine oocytes by enhancing the expansion of CCs [17].

IL-7, a cytokine primarily secreted by stromal cells in bone marrow and thymus, plays a pivotal role in cellular differentiation of lymphocytes [18]. IL-7 binds to the IL-7 receptor complex that consists of the IL-7Rα chain (IL-7R) and the common cytokine γ chain (γc) [19]. The IL-7R binds explicitly to IL-7 and leads to Janus kinase 1 (JAK1) activity, a receptor related to tyrosine Janus kinases, whereas the γc activates Janus kinase 3 (JAK3) [20]. Several signaling pathways that are involved in proliferation, survival, cell cycling, and metabolism, are affected when IL-7 binds to these receptors [21]. In naïve and memory T cells, IL-7 acts as an essential factor for survival [22] and homeostatic proliferation by downregulating apoptotic activity and upregulating growth activity [23]. In the reproductive system, oocyte-secreted IL-7 has been shown to act on granulosa cells as a survival factor by inhibiting apoptosis and enhanced oocyte maturation in rats [24]. In addition, IL-7 can potentially enhance the developmental competence of oocytes by stimulating proliferation of mouse CCs [25]. Recent research reported that low IL-7 concentrations increased favorable intracellular reactive oxygen species (ROS) levels and significantly improved oocyte maturation rate by downregulating the apoptotic process in sheep [26].

Although these studies have reported a role for IL-7 in other species, the function of IL-7 in the porcine reproductive system, including its effect on oocytes and CCs, has not been investigated. Therefore, we hypothesized that supplementation with IL-7 during IVM may enhance the oocyte quality by replenishing insufficient intracellular signaling in the in vitro environment. In this study, we investigated whether IL-7 is effective in both porcine IVM and the subsequent pre-implantation embryonic development following parthenogenetic activation (PA).

## 2. Materials and Methods

### 2.1. Chemicals and Reagents

Recombinant human IL-7 was purchased from PeproTech (London, UK). Unless otherwise stated, all chemicals and reagents used in the present study were purchased from Sigma-Aldrich (St. Louis, MO, USA).

### 2.2. Measurement of IL-7 in Porcine Follicular Fluid (FF)

Follicular fluid was collected from different ovary pairs of prepubertal three-way cross pigs (mixed Yorkshire, Landrace, and Duroc breeds) obtained in a local abattoir. Porcine follicles were aspirated from three groups according to their diameters (small (1–2 mm), medium (3–7 mm), or large (≥8 mm)) [27]. All collected porcine FF were centrifuged at 1000 rpm at 4 °C for 20 min to eliminate debris and blood. Supernatants were separately filtered through 1.2 μm syringe filters (Sartorius Stedim Biotech, Aubagne, France) and then frozen at −80 °C until analysis. Concentrations of IL-7 in porcine FF were measured using an ELISA kit (Fine Test, Wuhan, China), according to the manufacturer’s instructions. All ELISA experiments were performed in quadruplicate, and a mean value was used to determine the IL-7 concentration in porcine FF.

### 2.3. Oocyte Collection and IVM

Porcine ovaries were obtained from a local abattoir and transported to the laboratory within 1 h in 0.9% NaCl at 37 °C. Thereafter, cumulus-oocyte-complexes (COCs) were aspirated into 15 mL conical tubes from antral follicles of 3–7 mm in size using an 18-gauge needle and a 10 mL disposable syringe. The debris was allowed to settle at the bottom of the tube at 37 °C for 5 min. Subsequently, the porcine FFs were removed and the sediments were resuspended twice in HEPES-buffered Tyrode’s medium containing 0.05% (wt/vol) polyvinyl alcohol (TLH-PVA). The COCs surrounded with intact compact CCs layers and with evenly granulated cytoplasm were chosen using a stereomicroscope for IVM and then washed twice in TLH-PVA. After washing once in IVM medium, approximately 50–60 arbitrarily selected COCs were cultured in IVM medium in a four-well Nunc dish (Nunc, Roskilde, Denmark). IVM medium (TCM-199; Invitrogen Corporation, Carlsbad, CA, USA) supplemented with 0.6 mM cysteine, 0.91 mM sodium pyruvate, 10 ng/mL epidermal growth factor, 75 μg/mL kanamycin, 1 μg/mL insulin, and 0.1% (wt/vol) PVA was added to each well. The IVM process occurred over 42 h. Collected COCs were incubated with 10 IU/mL equine chronic gonadotropin (eCG) and 10 IU/mL human chorionic gonadotropin (hCG) (Intervet, Boxmeer, Netherlands) at 39 °C in a humidified 5% CO_2_ atmosphere for the first 22 h. Thereafter, COCs were cultured in a hormone-free maturation medium for the remaining 20 h. During the entire IVM period, IL-7 was added to the media at concentrations of 0 (control), 0.1, 1, and 10 ng/mL for each group. The concentration of IL-7 was decided based on a previous IL-7 study in rat granulosa cells [24].

### 2.4. Assessment of Nuclear Maturation

Oocytes from COCs were obtained by mechanically denuding surrounding CCs using 0.1% hyaluronidase after IL-7 supplementation post-IVM. The denuded oocytes were washed twice in TLH-PVA medium and immediately transferred into 30 μL TLH-PVA containing 5 μg/mL Hoechst-33342, and then stained in the dark for 10 min to assess the nuclear maturation rates. The stained oocytes were observed under a fluorescence microscope (Nikon Corp., Tokyo, Japan) with a UV filter (370 nm). Oocytes from each group were classified into germinal vesicle (GV), metaphase I (MI), anaphase-telophase I (AT), and metaphase II (MII) stages according to the classification by Naito and Toyoda [28].

### 2.5. Measurement of Intracellular GSH and ROS Levels

Denuded oocytes of MII-stage were collected using 0.1% hyaluronidase after 42 h IVM to evaluate the intracellular GSH and ROS levels. The GSH and ROS levels were determined using methods according to You et al. [29]. Briefly, CellTracker Blue 4-chloromethyl-6,8-difluoro-7-hydroxycoumarin (CMF_2_HC; indicated by blue fluorescence; Invitrogen) and 2′,7′-dichlorodihydrofluorescein diacetate (H_2_DCFDA; indicated by green fluorescence; Invitrogen) were used to measure GSH and ROS in the oocyte cytoplasm, respectively. Twenty oocytes were placed in 30 μL TLH-PVA supplemented with 10 μM CMF_2_HC or 10 μM H_2_DCFDA and incubated in the dark for 30 min. Thereafter, the stained oocytes were washed thrice with TLH-PVA and placed into a 8 μL drop of TLH-PVA, which was subjected to fluorescence microscopy using an epifluorescence microscope (TE300; Nikon) with UV filter (370 nm for GSH and 460 nm for ROS). Adobe Photoshop CS6 was used to examine the fluorescence intensity of oocytes and normalized to control oocytes. The independent experiment was repeated three times (GSH samples, N = 60; ROS samples, N = 60).

### 2.6. Gene Expression Analysis Using Real-Time qPCR

Matured oocytes and CCs were sampled from 50–60 COCs from each group by gently pipetting with 0.1% hyaluronidase after the 42 h IVM. Denuded oocytes of MII-stage and their respective CCs were sampled into 1.5 mL microfuge tubes and frozen at −80 °C until assayed. The gene expression analysis was performed using real-time qPCR for 13 specific genes associated with different functions, such as apoptosis: BCL2 associated X (*BAX*), BCL2 like 1 (*BCL2L1*), and caspase-3 (*CASP3*); mitochondrial-related genes: transcription factor A (*TFAM*) and NADPH oxidase 4 (*NOX4*); antioxidant-related genes: glutathione-disulfide reductase (*GSR*) and peroxiredoxin 1 (*PRDX1*); IL-7 associated genes: phosphoinositide-3-kinase regulatory subunit 1 (*PIK3R1*), AKT serine/threonine kinase 1 (*AKT1*), and solute carrier family 2 member 1 (*SLC2A1*; *GLUT1*); developmental competence-related genes: proliferating cell nuclear antigen (*PCNA*), KH domain containing 3 like (*KHDC3L*; *Filia*), nucleophosmin/nucleoplasmin 2 (*NPM2*), hyaluronan synthase 2 (*Has2*), and TNF alpha induced protein 6 (*TNFAIP6*). The mRNA expression levels of *BAX*, *BCL2L1*, *CASP3*, *TFAM*, *NOX4*, *GSR*, *PRDX1*, *PIK3R1*, *AKT1*, *GLUT1*, *PCNA*, *Has2*, and *TNFAIP6* were measured in CCs, and those of *BAX*, *BCL2L1*, *CASP3*, *NOX4*, *GSR*, *PRDX1*, *PIK3R1*, *AKT1*, *GLUT1*, *PCNA*, *Filia*, and *NPM2* were measured in oocytes. All primer sequences are provided in Table S1.

Total RNA was isolated from stored CCs and oocytes using TRIzol reagent (TaKaRa Bio, Inc., Otsu, Shiga, Japan) according to the manufacturer’s protocol. Complementary DNA (cDNA) was prepared from 1 μg of total RNA using Reverse Transcription Master Premix (Elpis Bio, Inc., Daejeon, Korea). In total, 1 μg of the synthesized cDNA was amplified using 2× SYBR Premix Ex Taq (Takara Bio, Inc.) and specific primers (5 pmol) by real-time qPCR (CFX96 real-time qPCR cycler (Bio-Rad, Hercules, CA, USA). The cycling parameters were as follows: 95 °C for 5 min, followed by 40 cycles of 15 s at 95 °C, 15 s at 56 °C and 30 s at 72 °C. The expression of each target gene in CCs and oocytes was quantified relative to a housekeeping gene, glyceraldehyde 3-phosphate dehydrogenase (*GAPDH*) and 18S ribosomal RNA (*RN18S*). The relative quantification was determined by comparing the threshold cycle (Ct) at constant fluorescence intensity. The relative mRNA expression (R) was calculated using the equation, R = 2^−[ΔCt sample − ΔCt control]^. The R values were normalized using *GAPDH* for cumulus cells, and *RN18S* for oocytes. The mean of three replicates was analyzed for statistical analysis.

### 2.7. Parthenogenetic Activation and In Vitro Culture of Porcine Embryos

After IVM, CCs were removed from oocytes as described above. The MII-stage oocytes from each group were selected to conduct PA as described by Jeon et al. [30]. The oocytes were washed twice in activation solution containing 280 mM mannitol, 0.01 mM CaCl_2_ and 0.05 mM MgCl_2_. The activation chamber connecting electrodes was filled with 2 mL of activation solution. The oocytes were placed in the chamber and then activated with two direct-current pulses of 120 V/mm for 60 μs. After PA, oocytes were transferred to IVC medium [31] supplemented with 5 μg/mL of cytochalasin B for 4 h under a humidified atmosphere of 5% CO_2_, 5% O_2_ and 90% N_2_. After incubation, the activated embryos were washed thrice in the IVC medium, placed for seven days in 25 μL droplets of IVC medium (10 gametes per drop) covered with mineral oil, and then cultured under the same atmospheric conditions. The culture media were refreshed at 48 h (Day 2) and 96 h (Day 4, with 10% FBS) after PA.

### 2.8. Evaluation of Developmental Competence and Total Cell Count

Day 0 was regarded as the day on which PA was initiated. Day 2 after PA, the cleavage formation was analyzed and embryos were categorized into three groups (2–3 cells, 4–6 cells and 7–8 cells). Blastocyst formation was assessed at seven days post PA and the blastocysts were categorized into three groups according to their morphology (early, expanded, and hatched), as reported in a previous study [8]. To calculate the total cell number of blastocysts at Day 7, the blastocysts were washed in TLH-PVA and fixed in 4% paraformaldehyde in PBS-PVA and stained for 5 min using 5 μg/mL Hoechst-33342. Next, the blastocysts from each group were transferred to a drop of 100% glycerol on glass slides and gently covered with a coverslip. The stained blastocysts were observed using a fluorescence microscope (Nikon, Tokyo, Japan) at 400× magnification. The experiment was repeated three times.

### 2.9. Statistical Analysis

Each experiment was repeated at least three times. The COCs used in each experiment were collected in the same abattoir on the same day and then were randomly used in each group. The rates of cleavage and blastocyst formation from the activated embryos were analyzed on the same day. Statistical analysis was conducted using SPSS 12.0 (SPSS, Inc., Chicago, IL, USA). Percentage data (rates of nuclear maturation and embryonic development) and average data (ELISA, intracellular GSH and ROS levels in oocytes and total cell number in blastocyst) were analyzed using Duncan’s multiple range test after one-way ANOVA. The data are presented as the mean or the mean ± standard error of the mean (SEM). The values from the ELISA experiments are presented as the mean ± standard deviation (SD). Values of *p* < 0.05 were considered statistically significant.

## 3. Results

### 3.1. Detection of IL-7 in Porcine FF

ELISA was performed to determine the concentration of IL-7 in porcine FFs at each ovarian follicle size (small, medium, and large). The maturation medium, M199, was used as a negative control. IL-7 was detected in all porcine FFs obtained from different follicle sizes. Interestingly, the concentration of IL-7 in the medium FF group (64.2 ± 39.2 pg/mL) was significantly higher than that of the small FF group (6.8 ± 5.0 pg/mL) (*p* < 0.05). There was no significant difference in the large FF group (44.0 ± 22.8 pg/mL) compared to other groups (Table 1).

### 3.2. Effect of IL-7 Supplementation during IVM on Oocyte Nuclear Maturation

To evaluate the effect of IL-7 treatment on the nuclear maturation of porcine oocytes during IVM, matured oocytes were evaluated at stages GV, MI, AT, or MII. After 42 h of IVM, the 1 ng/mL IL-7-supplemented group (97.3%) showed a significantly higher (*p* < 0.05) MII rate compared with the control (91.6%). However, no significant differences were observed in other IL-7-treated groups (0.1 and 10 ng/mL group: 92.2 and 92.1%) compared with the control (Table 2).

### 3.3. Effect of IL-7 Supplementation during IVM on Cytoplasmic Maturation

**Figure 1:**
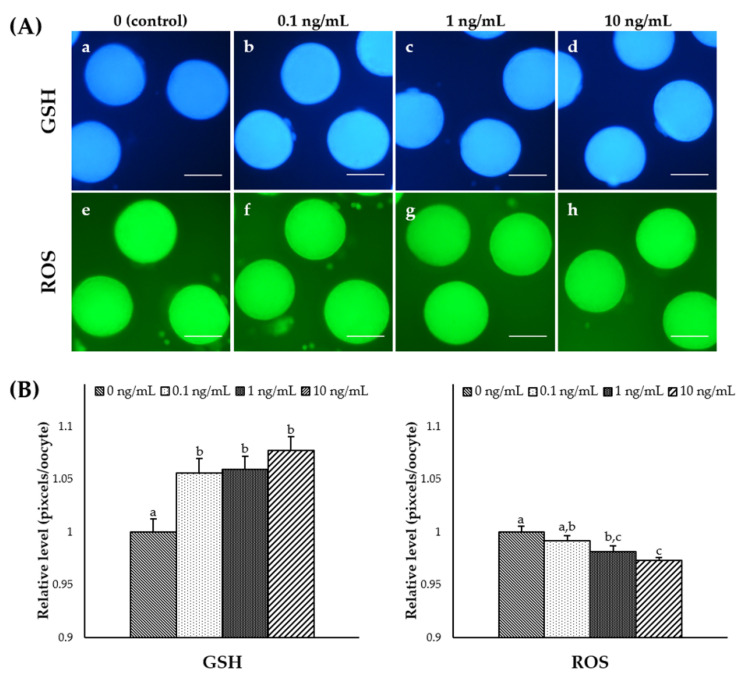
Epifluorescence photomicrograph images of in vitro matured porcine oocytes. (**A**) Oocytes stained with Cell Tracker Blue (a–d) and H_2_DCFDA (e–h) to detect intracellular levels of glutathione (GSH) and reactive oxygen species (ROS), respectively. Metaphase II (MII) oocytes derived from the control in vitro maturation (IVM) system and the IVM system supplemented with various concentrations of interleukin-7 (IL-7). (**B**) The relative levels of intracellular GSH and ROS levels in in vitro matured porcine oocytes treated with IL-7 during IVM. The bars with different letters (a–c) are significantly different (*p* < 0.05). GSH samples, N = 60; ROS samples, N = 60. The experiment was replicated three times. Scale bar: 100 μm.

To assess the effects of IL-7 supplementation on cytoplasmic maturation during IVM, MII stage oocytes from each group were selected and stained as described above (Figure 1A). The intracellular GSH levels were significantly increased (*p* < 0.05) in oocytes from all IL-7-treated groups compared to the control group (Figure 1B). The 1 and 10 ng/mL IL-7-treated groups showed significantly (*p* < 0.05) lower intracellular ROS levels than the control group (Figure 1B). In this study, the IL-7 supplemented during IVM significantly affected intracellular GSH and ROS levels in matured oocytes.

### 3.4. Effect of IL-7 Supplementation on Gene Expression Levels in CCs and Oocytes during IVM

**Figure 2:**
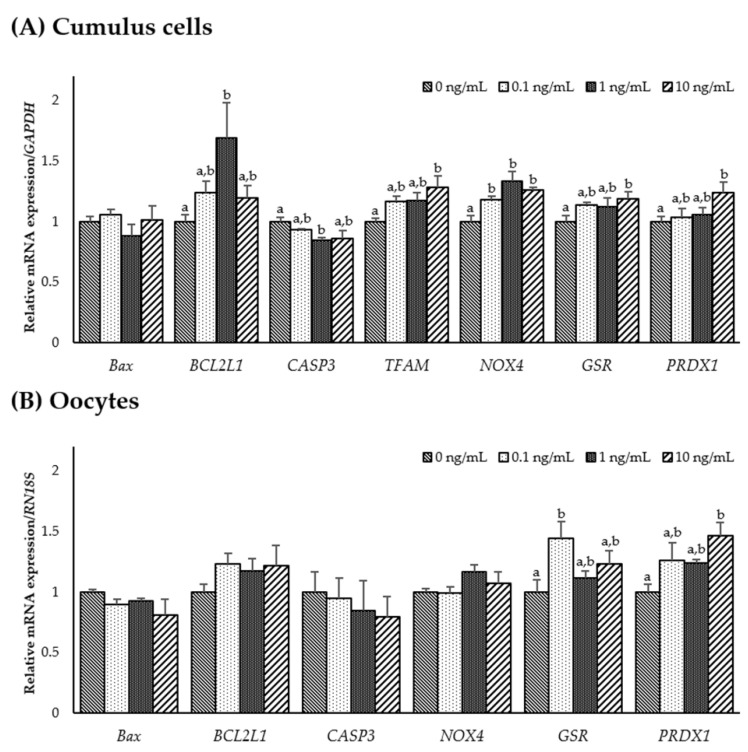
The mRNA expression levels (mean ± SEM) of apoptosis-, mitochondrial-, and antioxidant-related genes. Levels of *Bax*, *BCL2L1*, *CASP3*, *TFAM*, *NOX4*, *GSR1*, and *PRDX1* assessed in groups of cumulus cells (**A**) and levels of *Bax*, *BCL2L1*, *CASP3*, *NOX4*, *GSR1*, and *PRDX1* assessed in groups of oocytes (**B**) supplemented with various concentrations of interleukin-7 after in vitro maturation. The experiment was replicated three times. The bars with different letters (a, b) are significantly different (*p* < 0.05).

To examine the effect of IL-7 on the expression of apoptosis-, mitochondrial-, and antioxidant-related genes, the mRNA expression levels of *Bax*, *BCL2L1*, *CASP3*, *TFAM*, *NOX4*, *GSR*, and *PRDX1* were assessed in CCs and oocytes. As shown in Figure 2A, the CCs supplemented with 1 ng/mL IL-7 showed significantly higher anti-apoptotic gene *BCL2L1* levels and lower pro-apoptotic gene *CASP3* levels than the control group (*p* < 0.05). In the mitochondrial-related genes, the transcription levels of *TFAM* were significantly increased in the CCs treated with 10 ng/mL IL-7 compared with control (*p* < 0.05). However, the *TFAM* transcript levels in oocytes were too low to be accurately quantified. In addition, *NOX4* transcript levels were significantly higher in all IL-7-treated CCs than in the control group (*p* < 0.05). Levels of the antioxidant-related genes *GSR* and *PRDX1* were significantly increased in the CCs treated with 10 ng/mL IL-7 compared with controls (*p* < 0.05). Further, oocytes supplemented with 0.1 ng/mL IL-7 displayed significantly higher (*p* < 0.05) transcript levels of *GSR* than the control group (Figure 2B). *PRDX1* mRNA levels appeared significantly increased in oocytes treated with 10 ng/mL IL-7 (*p* < 0.05).

**Figure 3:**
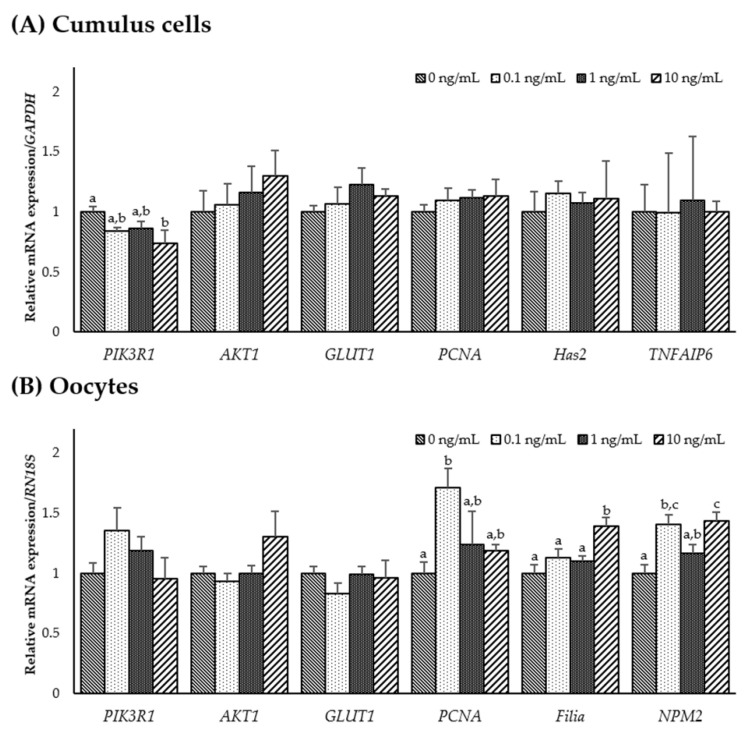
The mRNA expression levels (mean ± SEM) of interleukin-7 (IL-7)-associated and developmental competence-related genes. Levels of *PIK3R1*, *AKT1*, *GLUT1*, *PCNA*, *Has2*, and *TNFAIP6* assessed in groups of cumulus cells (**A**) and levels of *PIK3R1*, *AKT1*, *GLUT1*, *PCNA*, *Filia*, and *NPM2* assessed in groups of oocytes (**B**) supplemented with various concentrations of IL-7 after in vitro maturation. The experiment was replicated three times. The bars with different letters (a–c) are significantly different (*p* < 0.05).

To investigate the role of IL-7 on the expression of IL-7-associated and developmental competence-related genes, mRNA expression levels of *PIK3R1*, *AKT1*, *GLUT1*, *PCNA*, *Has2*, *TNFAIP6*, *Filia*, and *NPM2* were determined in CCs and oocytes from each group. As shown in Figure 3A, transcription levels of *PIK3R1* significantly decreased in CCs supplemented with 10 ng/mL IL-7 compared to the control (*p* < 0.05). However, no significant difference was observed in *PIK3R1*, *AKT1*, and *GLUT1* transcript levels in IL-7-supplemented oocytes compared with the control group. In developmental competence-related genes, *PCNA* transcript levels were significantly higher in 0.1 ng/mL IL-7-treated oocytes than in the control (*p* < 0.05). Transcript levels of *Filia* and *NPM2* were significantly increased (*p* < 0.05) in oocytes treated with 0.1 or 10 ng/mL IL-7 compared with the control group (Figure 3B).

### 3.5. Effect of IL-7 Supplementation in IVM Media on Developmental Potential after PA

**Figure 4:**
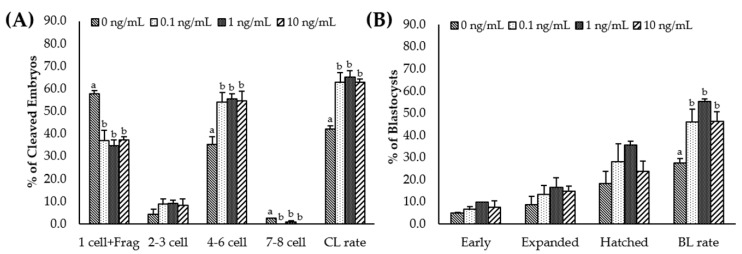
Effect of interleukin-7 supplementation on the cleavage pattern during in vitro maturation (**A**) and the blastocyst formation pattern (**B**) of the parthenogenetic activation (PA) embryo. Within each end point, bars with different letters (a, b) differ significantly (*p* < 0.05) for different concentrations of IL-7. CL, cleavage; BL, blastocyst. The cleavage and blastocyst rates were evaluated on day 2 and 7 after PA, respectively. The experiment was replicated three times.

After PA, cleavage and blastocyst rates were significantly higher (*p* < 0.05) in all IL-7-treated groups than in the control group (Table 3, Figure S1). In addition, the total cell number in the blastocyst was significantly (*p* < 0.05) increased in the 1 ng/mL IL-7-treated group compared to the control (Table 3). The cleavage patterns differed in IL-7-supplemented groups. As shown in Figure 4A, 4–6 cell cleavage rates of the PA embryos were significantly increased in all IL-7-supplemented groups (*p* < 0.05). The 1-cell and fragmentation group and 7–8 cell cleavage rates were significantly lower in all IL-7-treated groups than in the control group (*p* < 0.05).

## 4. Discussion

Bidirectional communication between the oocyte and its surrounding somatic cells is essential for follicle development, oocyte maturation, and acquisition of developmental competence for oocytes in mammals [13,32]. OSFs, such as GDF9 and BMP15, are involved in this interaction [33]. IL-7 is an OSF detected in human FF [34,35]. However, the physiological function of IL-7 during oogenesis and folliculogenesis is still unknown. This is the first study to demonstrate the presence of IL-7 in porcine FF. Notably, supplementation with IL-7 during IVM exerted beneficial effects on nuclear maturation and improved cytoplasmic maturation of porcine oocytes by upregulating antioxidant-related genes. IL-7 also enhanced the subsequent developmental potential of PA porcine embryos. Further, expression levels of various genes were significantly changed in CCs and matured oocytes post-IL-7 treatment.

Cytokines are essential for successful progression during folliculogenesis, such as primary-to-antral follicle transition [36]. The intraovarian/perifollicular cytokine environment is closely related to oocyte quality and viability [37]. This study showed that the cytokine IL-7 is present in porcine ovarian FF. Furthermore, the concentration of IL-7 in medium follicles (3–7 mm) is significantly higher than that observed in small follicles (1–2 mm). In patients with abnormal ovarian follicles, a lower IL-7 level in FF is correlated with lower embryo quality and lower in vitro fertilization outcome [34]. As previously reported, oocytes collected from small follicles (≤ 3 mm) reveal inadequate cytoplasmic maturation due to lower levels of stored mRNA and proteins related to developmental competence [38,39]. Hence, higher IL-7 levels found in medium follicles provide evidence of its role in porcine follicle development.

Oocyte meiotic maturation is an essential process during in vitro production (IVP) of embryos and determines whether an oocyte is competent to undergo fertilization and embryogenesis. This process contains developmental programs such as nuclear and cytoplasmic maturation. For the successful IVM of oocytes, the oocytes must undergo synchronization between nuclear and cytoplasmic maturation [40,41]. Previous studies reported that IL-7 elevates nuclear maturation of pre-ovulatory oocytes in rats and sheep [24,26]. Consistently, the 1 ng/mL IL-7 treatment significantly increased the number of porcine oocytes at the MII stage. This study suggests that IL-7 supplementation during IVM can positively affect the nuclear maturation of porcine oocytes.

After IVM, GSH levels in oocytes are regarded as an indicator of oocyte cytoplasmic maturation [42]. Further, GSH plays a vital role in protecting the cell against ROS toxic activity [43]. Results indicate that IL-7 supports the production of intracellular GSH and suppresses ROS levels in matured oocytes. ROS often induce cellular damage, leading to apoptosis, and cause DNA breakdown due to the formation of free radicals [44,45]. ROS play a concentration-dependent role in meiotic resumption of the oocyte during mammalian reproduction—lower ROS concentration assists meiotic resumption, whereas a higher ROS level causes meiotic cell cycle arrest and apoptosis. Therefore, an adequate balance is vital for oocyte quality [45]. In human T-cell acute lymphoblastic leukemia cells, IL-7-induced generation of ROS is reportedly involved in PI3K-AKT signaling, which aids survival [46]. In ovine studies, the appropriate treatment of IL-7 increases favorable intracellular ROS levels in matured oocytes [26]. In contrast, the results of this study indicate a decrease in ROS levels in mature oocytes, which is thought to be due to differences between species and cell source. Consistent with GSH and ROS results, the 10 ng/mL IL-7-supplemented oocytes and CCs displayed a significant up-regulation of *GSR* and *PRDX1* mRNA. The H_2_O_2_ produced by ROS can be converted to H_2_O by PRDX1 and GSH [47]. GSH homeostasis is regulated by GSR, which catalyzes the reduction of glutathione disulfide (GSSG) to the sulfhydryl form GSH [48]. Therefore, the GSH increase is likely to be exerted by increased expression of *GSR* and *PRDX1* transcript levels by IL-7, along with its direct antioxidant effect to improve the cytoplasmic maturation during porcine IVM.

Mammalian studies reported that an increase in GSH and decrease in ROS levels in oocytes during IVM are related to developmental competence and affect female reproductive outcome [49,50,51]. Consistent with the effects on GSH and ROS, the IL-7-treated groups exhibited significantly increased mRNA expression levels of the developmental-related genes *PCNA*, *Filia*, and *NPM2* in matured oocytes. It was reported that PCNA determines the fate of the oocyte by increasing its expression in oocytes during the initiation of primordial follicle development [52]. Furthermore, accumulation of *Filia* and *NPM2* mRNA, as maternal effect genes, in oocytes activates the embryonic genome and are involved in oocyte maturation, fertilization, and early embryonic development [53,54]. However, *Has2* and *TNFAIP6* transcript levels, as cumulus expansion-related genes [55], did not affect the CC expansion upon IL-7 treatment. Consistent with a previous report, IL-7 does not appear to be involved in CC expansion [25]. In the PA embryonic development analysis, the groups treated with IL-7 displayed significantly increased cleavage and blastocyst formation rates. Therefore, IL-7 supplementation during IVM improved embryonic development after PA by upregulating developmental competence genes in porcine oocytes.

Several previous studies have mentioned the importance of communication between oocytes and CCs to acquire oocyte developmental competence [13,56,57]. In particular, increased apoptosis in CCs causes detrimental nuclear maturation in oocytes and reduces the fertilization rate [58]. IL-7 reportedly suppresses precursor B cell apoptosis by providing a survival factor via the BCL-2 family proteins [59]. Moreover, IL-7 decreases apoptosis of granulosa cells via suppression of caspase 3/7 [24]. As previously reported, *CASP3* and *caspase-7* transcript levels were increased in the CCs of patients with polycystic ovary syndrome, which can lead to higher apoptosis in these cells and negatively affect embryo quality [60]. This study observed that transcript levels of *BCL2L1* increased, while that of *CASP3* decreased in CCs treated with 1 ng/mL IL-7, compared with controls. The caspase-3 cascade, a mitochondrial apoptotic signaling pathway, is regulated by cytochrome c released from mitochondria. Mitochondrial cytochrome c is repressed by the anti-apoptotic BCL-2 protein [61]. Therefore, this result indicated that IL-7 may inhibit mitochondrial-associated apoptosis in porcine CCs. However, it is unclear how IL-7 increased the transcript levels of *BCL2L1* in porcine CCs, and further study is necessary. Furthermore, this study found that CCs treated with IL-7 significantly increased transcript levels of *TFAM* and *NOX4*. The production of *TFAM* mRNA is related to mitochondrial DNA transcription and supports mitochondrial respiratory function [62,63]. In addition, NOX4 is located in the mitochondria and produces ROS [64]. It was previously reported that ROS induced by mitochondrial respiration and NADPH oxidase contributes to the survival of T-cell acute lymphoblastic leukemia cells by being associated with IL-7-mediated signaling [46]. These results indicated that IL-7 may potentially enhance oocyte developmental competence by interacting with mitochondrial-related apoptosis and survival signals in porcine CCs. Therefore, further study is necessary to investigate the potential for mitochondrial function following IL-7 supplementation during porcine IVM.

## 5. Conclusions

In conclusion, for the first time, the present study demonstrated that IL-7 is present in porcine FF, using ELISA. The present study showed that IL-7 supplementation enhances porcine oocyte meiotic maturation by simultaneously improving nuclear maturation and cytoplasmic maturation due to the antioxidant effect. In particular, the enhanced oocyte quality improved the subsequent developmental potential of porcine embryos after PA. Furthermore, IL-7 treatment during IVM may improve the developmental competence of oocytes by inhibiting mitochondrial-related apoptosis and improving survival in porcine CCs. These findings may provide further insight into the effects of IL-7 on follicular development or oocyte meiotic maturation and might aid in enhancement of porcine IVM system and related techniques.

## Figures and Tables

**Table 1:** The concentration of interleukin-7 (IL-7) in porcine follicular fluids from different follicle sizes (small, medium, and large).

Cytokine	Size of Follicles (n* = 25)			M199
	Small (1–2 mm)	Medium (3–7 mm)	Large (≥8 mm)	
IL-7 (pg/mL)	6.8 ± 5.0 ^a^	64.2 ± 39.2 ^b^	44.0 ± 22.8 ^a,b^	ND

The data shown are mean ± SD from three replicates. n* Number of follicles. ^a,b^ Values with different superscripts within a row differ significantly (*p* < 0.05). ND: not detected.

**Table 2:** Effect of interleukin-7 (IL-7) supplementation during in vitro maturation on nuclear maturation.

IL-7 Concentration (ng/mL)	No. of Oocytes Cultured for Maturation	Mean ± SEM (%) Oocytes at the Stage of			
		Germinal Vesicle	Metaphase I	Anaphase and Telophase I	Metaphase II
0	192	3.2 ± 1.4	1.6 ± 0.5	3.6 ± 1.8	91.6 ± 1.8 ^a^
0.1	179	2.2 ± 1.5	1.7 ± 0.6	3.9 ± 1.1	92.2 ± 1.0 ^a^
1	188	0.6 ± 0.6	0.5 ± 0.5	1.6 ± 0.5	97.3 ± 0.5 ^b^
10	191	2.7 ± 1.3	2.1 ± 0.8	3.2 ± 0.6	92.1 ± 1.1 ^a^

The experiment was replicated four times. ^a,b^ Values with different superscripts within a column differ significantly (*p* < 0.05).

**Table 3:** Effect of interleukin-7 (IL-7) supplementation during in vitro maturation on developmental potential after parthenogenetic activation.

IL-7 Concentration (ng/mL)	No. of Embryos Cultured, N*	No. (%) of Embryos Developed to		Total Cell Number in Blastocyst (n**)
		≥ 2-Cell	Blastocyst	
0	116	49 (42.3 ± 1.4) ^a^	32 (27.6 ± 2.0) ^a^	90.1 ± 6.9 (32) ^a^
0.1	114	72 (62.9 ± 4.3) ^b^	53 (46.0 ± 5.8) ^b^	89.4 ± 5.1 (53) ^a^
1	121	79 (65.3 ± 2.6) ^b^	67 (55.4 ± 1.1) ^b^	114.7 ± 5.8 (67) ^b^
10	121	76 (62.8 ± 1.5) ^b^	56 (46.3 ± 4.5) ^b^	98.0 ± 5.1 (56) ^a^

N*: The experiment was repeated three times. n**: Number of blastocysts examined. ^a,b^ Values with different superscripts within a column differ significantly (*p* < 0.05).

## Data Availability

The data presented in this study are available on request from the corresponding author.

## Supplementary Materials

The following are available online at https://www.mdpi.com/2076-2615/11/3/741/s1, Table S1: Primers list for real-time qPCR, Figure S1: Representative morphology image of blastocysts from each group after Day 7 of PA.
