# Supplementary material for: Effect of Interleukin-7 on In Vitro Maturation of Porcine Cumulus-Oocyte Complexes and Subsequent Developmental Potential after Parthenogenetic Activation
Source: Animals (Basel). 2021 Mar 8;11(3):741. doi: 10.3390/ani11030741 (PMC8001781; doi:10.3390/ani11030741)
Supplement: Supplementary file 1 [file animals-11-00741-s001.pdf]

Table S1. Primers list for real-time qPCR.

mRNA	Primer Sequences	Tm (°C)	Product Size (bp)	GenBank Accession Number
GAPDH	F: 5'-GTCGGTTGTGGATCTGACCT-3'	59	207	NM_001206359.1
	R: 5'-TTGACGAAGTGGTCGTTGAG-3'	58		
RN18S	F: 5'-CGCGGTTCTATTTTGTTGGT-3'	57	219	NR_046261.1
	R: 5'-AGTCGGCATCGTTTATGGTC-3'	58		
BAX	F: 5'-TGCCTCAGGATGCATCTACC-3'	59	199	XM_013998624.2
	R: 5'-AAGTAGAAAAGCGCGACCAC-3'	58		
BCL2L1	F: 5'-AATGACCACCTAGAGCCTTG-3'	56	182	NM_214285.1
	R: 5'-GGTCATTTCCGACTGAAGAG-3'	55		
CASP3	F: 5'-CGTGCTTCTAAGCCATGGTG-3'	59	186	NM_214131.1
	R: 5'-GTCCCACTGTCCGTCTCAAT-3'	59		
TFAM	F: 5'-TGGTCCATCACAGGTAAAGC-3'	57	209	NM_001130211.1
	R: 5'-CCTCAGTGTCTTTCTTTGCTG-3'	57		
NOX4	F: 5'-CTTCCTCCATCCAGTCAAGA-3'	56	185	XM_003357234.4
	R: 5'-TCCAGCAGGGTGTTGAGTAT-3'	58		
GSR	F: 5'-TGGGCTCTAAGACGTCACTG-3'	59	106	XM_003483635.4
	R: 5'-TCTATGCCAGCATTCTCCAG-3'	57		
PRDX1	F: 5'-CTTGATATCAGACCCCAAGC-3'	55	187	XM_021096742.1
	R: 5'-GAACTGGAAGGCCTGAACTA-3'	56		
PIK3R1	F: 5'-CCACTACCGGAATGAATCTC-3'	55	211	XM_021076847.1
	R: 5'-TTCCTGGGAAGTACGGGTAT-3'	57		
AKT1	F: 5'-CTACAACCAGGACCACGAGA-3'	58	208	NM_001159776.1
	R: 5'-CTCATACACATCCTGCCACA-3'	57		
GLUT1	F: 5'-TGGATGTCCTACCTGAGCAT-3'	57	192	XM_021096908.1
	R: 5'-CTCCACATACTGGAAGCACA-3'	57		
PCNA	F: 5'-CCTGTGCAAAAGATGGAGTG-3'	57	187	NM_001291925.1
	R: 5'-GGAGAGAGTGGAGTGGCTTTT-3'	59		
Filia	F: 5'-GCCCTACTGGTTTCACTCAG-3'	57	193	XM_021089533.1
	R: 5'-ATAAGGCCGTCCAAATATCA-3'	54		
NPM2	F: 5'-GCTCTGGACCTGTGTTCCTC-3'	60	220	NM_001195362.1
	R: 5'-GCTGCACTTGTCTGCTTCTG-3'	59		
Has2	F: 5'-TTACAATCCTCCTGGGTGGT-3'	58	199	NM_214053.1
	R: 5'-TCAAGCACCATGTCGTACTG-3'	58		
TNFAIP6	F: 5'- TCATAACTCCATATGGCTTGAAC-3'	56	396	NM_001159607.1
	R: 5'- TCTTCGTACTCATTTGGGAAGCC-3'	60		

F: Forward, R: Reverse, Tm (°C): Melting temperature

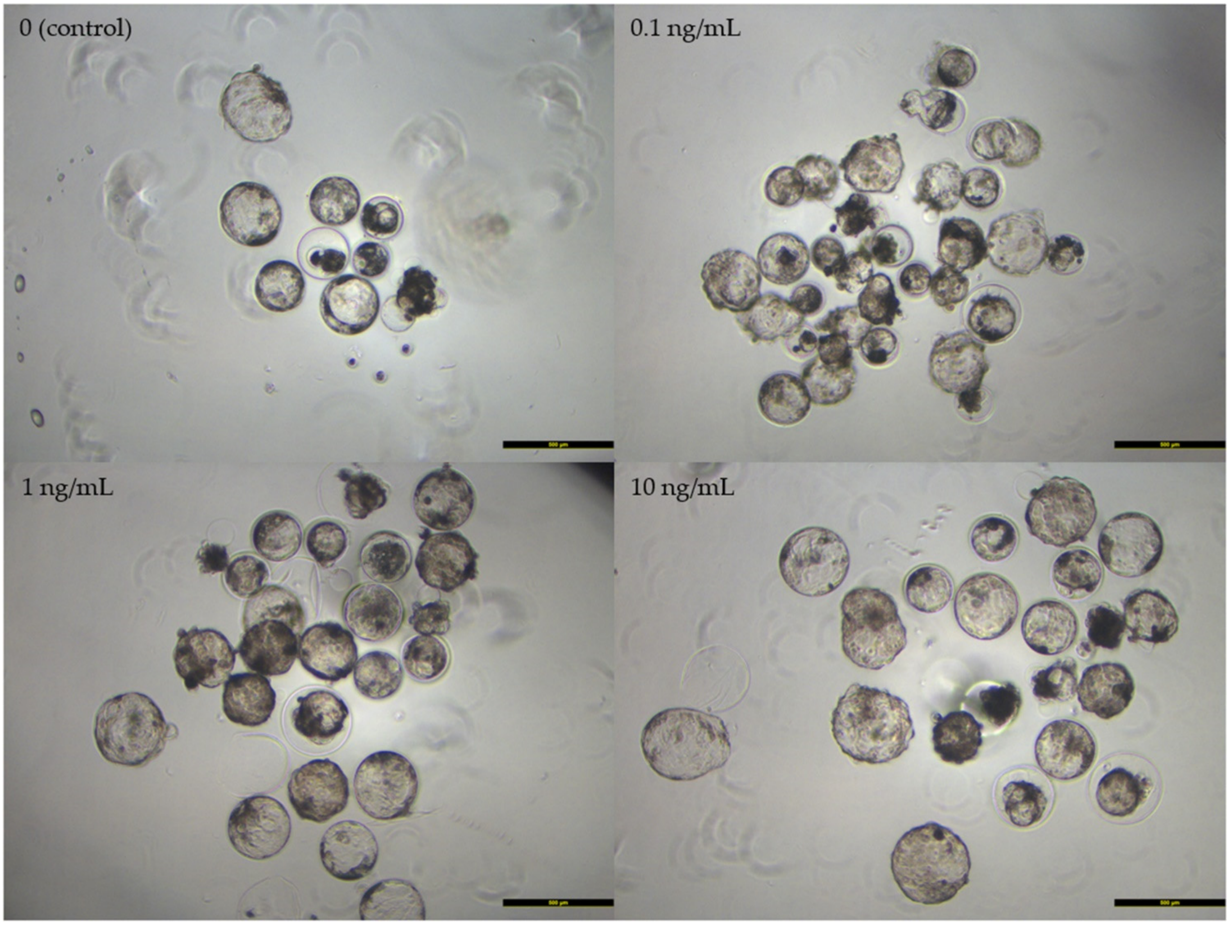

Figure S1. Representative morphology image of blastocysts from each group after Day 7 of PA. Scale bar: 500 μm
